# 62610 Effects of electronic verses paper based data capture in large multinational trials on time to complete, time to publication, participation and collaboration.

**DOI:** 10.1017/cts.2021.493

**Published:** 2021-03-30

**Authors:** Erika Davidoff, Nadina Jose, Barbara Tafuto

**Affiliations:** 1 Rutgers University Department of Biomedical Engineering; 2 Rutgers School of Health Professions

## Abstract

ABSTRACT IMPACT: My work evaluates the impact of electronic data capture/eSource on several aspects of clinical trial efficiency and scale, aiming to demonstrate how eSource can be used to improve the way we run clinical trials. OBJECTIVES/GOALS: Using eSource may increase the efficiency of data collection in clinical trials. However, adoption of eSource has been slow. We reviewed over 100 large multinational clinical trials to analyze how eSource use correlated with trial size, sponsor collaborations, time to complete, and time to publication. METHODS/STUDY POPULATION: We searched ClinicalTrials.gov for completed, interventional, Stage II-IV clinical trials with posted results and an uploaded study protocol document. This produced 3,962 trials. We identified all studies with over 1,200 participants and sites in multiple countries (or at least 100 sites in one country). After eliminating ten studies with duplicate protocols, we had a database of 123 trials. From the ClinicalTrials.gov listing, the study protocol, and any published papers, we determined the start, end, and publication dates, data collection protocol, sponsors and collaborators, and any reasons given for delays for each trial. We performed statistical tests comparing trial delay, participant and country count, and collaboration status (yes or no) between the two groups (eSource users and non-eSource users). RESULTS/ANTICIPATED RESULTS: Of our 123 trials, 60 (48.7%) used eSource, 48 (39.1%) used paper source documentation, and 15 (12.2%) used some combination. We found no statistically significant difference between eSource and non-eSource trials in terms of trial delay (p=0.43), time to publish (p=0.33), collaboration status (p=0.54), number of participants (p=0.36), or number of countries (p=0.12). However, our analysis was limited by what data was publically available. To investigate the effects of eSource on site efficiency, data accuracy, and data security, which are three major factors behind the FDA’s 2013 eSource recommendation, we would need access to proprietary information from trial sponsors. DISCUSSION/SIGNIFICANCE OF FINDINGS: The use of eSource in large multinational clinical trials is not correlated with a change in time to completion or publication nor a higher number of participants or countries. We aim to acquire proprietary data to further analyze the impacts of eSource on trial efficiency, data accuracy, and data security.

